# Inter-society consensus document on treatment and prevention of bronchiolitis in newborns and infants

**DOI:** 10.1186/1824-7288-40-65

**Published:** 2014-10-24

**Authors:** Eugenio Baraldi, Marcello Lanari, Paolo Manzoni, Giovanni A Rossi, Silvia Vandini, Alessandro Rimini, Costantino Romagnoli, Pierluigi Colonna, Andrea Biondi, Paolo Biban, Giampietro Chiamenti, Roberto Bernardini, Marina Picca, Marco Cappa, Giuseppe Magazzù, Carlo Catassi, Antonio Francesco Urbino, Luigi Memo, Gianpaolo Donzelli, Carlo Minetti, Francesco Paravati, Giuseppe Di Mauro, Filippo Festini, Susanna Esposito, Giovanni Corsello, on behalf of their respective Scientific Pediatric Societies

**Affiliations:** SIMRI-Società Italiana per le Malattie Respiratorie Infantili, Kragujevac, Italy; Women’s and Children’s Health Department, Unit of Pediatric Respiratory Medicine and Allergy, University of Padova, Via Giustiniani 3, 35128 Padova, Italy; SIN-Società Italiana di Neonatologia, Kragujevac, Italy; SICP-Società Italiana di Cardiologia Pediatrica, Kragujevac, Italy; AIEOP - Società Italiana di Ematologia e Oncologia Pediatrica, Kragujevac, Italy; AMIETIP - Accademia Medica Infermieristica di Emergenza e Terapia Intensiva Pediatrica, Kragujevac, Italy; FIMP - Federazione Italiana Medici Pediatri, Kragujevac, Italy; SIAIP - Società Italiana di Allergologia e Immunologia Pediatrica, Kragujevac, Italy; SICuPP - Società Italiana delle Cure Primarie Pediatriche, Kragujevac, Italy; SIEDP - Società Italiana di Endocrinologia e Diabetologia Pediatrica, Kragujevac, Italy; SIFC - Società Italiana per lo studio della Fibrosi Cistica, Kragujevac, Italy; SIGENP - Società Italiana Gastroenterologia Epatologia e Nutrizione Pediatrica, Kragujevac, Italy; SIMEUP - Società Italiana di Medicina di Emergenza ed Urgenza Pediatrica, Kragujevac, Italy; SIMGePeD - Società Italiana Malattie Genetiche Pediatriche e Disabilità Congenite, Kragujevac, Italy; SIMP - Società Italiana di Medicina Perinatale, Kragujevac, Italy; SINP - Società Italiana di Neurologia Pediatrica, Kragujevac, Italy; SIPO - Società Italiana Pediatria Ospedaliera, Kragujevac, Italy; SIPPS - Società Italiana di Pediatria Preventiva e Sociale, Kragujevac, Italy; SISIP - Società Italiana di Scienze Infermieristiche Pediatriche, Kragujevac, Italy; SITIP - Società Italiana di Infettivologia Pediatrica, Kragujevac, Italy; SIP-Società Italiana di Pediatria, Kragujevac, Italy

**Keywords:** Bronchiolitis, Respiratory syncytial virus, Prematurity, Bronchopulmonary dysplasia, Congenital heart diseases, Immunodeficiency, Oxygen therapy, Prevention, Prophylaxis

## Abstract

Acute bronchiolitis is the leading cause of lower respiratory tract infection and hospitalization in children less than 1 year of age worldwide. It is usually a mild disease, but some children may develop severe symptoms, requiring hospital admission and ventilatory support in the ICU. Infants with pre-existing risk factors (prematurity, bronchopulmonary dysplasia, congenital heart diseases and immunodeficiency) may be predisposed to a severe form of the disease.

Clinical diagnosis of bronchiolitis is manly based on medical history and physical examination (rhinorrhea, cough, crackles, wheezing and signs of respiratory distress). Etiological diagnosis, with antigen or genome detection to identify viruses involved, may have a role in reducing hospital transmission of the infection.

Criteria for hospitalization include low oxygen saturation (<90-92%), moderate-to-severe respiratory distress, dehydration and presence of apnea. Children with pre-existing risk factors should be carefully assessed.

To date, there is no specific treatment for viral bronchiolitis, and the mainstay of therapy is supportive care. This consists of nasal suctioning and nebulized 3% hypertonic saline, assisted feeding and hydration, humidified O_2_ delivery. The possible role of any pharmacological approach is still debated, and till now there is no evidence to support the use of bronchodilators, corticosteroids, chest physiotherapy, antibiotics or antivirals. Nebulized adrenaline may be sometimes useful in the emergency room. Nebulized adrenaline can be useful in the hospital setting for treatment as needed. Lacking a specific etiological treatment, prophylaxis and prevention, especially in children at high risk of severe infection, have a fundamental role. Environmental preventive measures minimize viral transmission in hospital, in the outpatient setting and at home. Pharmacological prophylaxis with palivizumab for RSV bronchiolitis is indicated in specific categories of children at risk during the epidemic period.

Viral bronchiolitis, especially in the case of severe form, may correlate with an increased incidence of recurrent wheezing in pre-schooled children and with asthma at school age.

The aim of this document is to provide a multidisciplinary update on the current recommendations for the management and prevention of bronchiolitis, in order to share useful indications, identify gaps in knowledge and drive future research.

## Aim of the document and general aspects

This intersociety consensus document intends to provide a multidisciplinary update for the National Health System (NHS), hospital and emergency department pediatricians, pediatricians within the national health system, physicians attending postgraduate schools, nurses and in general healthcare providers in the pediatric area, concerning the most up-to-date scientific evidence on treatment and prevention of bronchiolitis, with special focus on high risk children populations. The drafting of an intersociety document has for the first time been adopted as a strategy concerning the topic of bronchiolitis, and this document results from the cooperation of 20 Italian scientific pediatric societies. Intersociety documents are currently considered to be the most effective tool to reach all pediatric healthcare providers, to convey shared indications useful in clinical practice, to identify any gaps in current knowledge and to drive future research.

The workgroup performing the necessary literature research in drafting this recommendation document used the PubMed database (until October 2013). The workgroup commits to updating the document every 3 years.

Viral bronchiolitis is the most frequent lower respiratory tract infection in infants [1] and Respiratory Syncytial Virus (RSV) [2, 3] is the most common infecting agent; however, other respiratory viruses such as Rhinovirus (RV), Parainfluenza and Metapneumovirus (MPV) have been recently demonstrated to cause this disease, occasionally in the form of co-infection [4, 5]. Bronchiolitis is the leading cause of hospitalization in infants (with a peak of hospitalization at the age of 2 months), and a number of these children will require admission to intensive care units and mechanical ventilation. Bronchiolitis is associated with high mortality rates not only in developing countries, as this disease represents the main cause of death due to viral infection during the first year of life also in industrialized countries, with an incidence which is nine times higher than that of influenza virus infections [6]. Severe forms of the disease requiring hospitalization may be more frequent in children younger than 3 months of age or children with pre-existing risk factors such as prematurity, bronchopulmonary dysplasia, congenital heart diseases and immunodeficiency [2, 7, 8]. As regards long-term outcomes, about 30–40% of children with prior bronchiolitis-related hospitalizations will experience recurrent bronchospasm episodes all throughout adolescence and afterwards [9, 10].

There is no evidence of efficacy for numerous therapies commonly used when treating bronchiolitis (bronchodilators, steroids, antibiotics) [11], and supportive treatment (oxygen and hydration) still remains the recommended approach, as confirmed by leading international guidelines, such as the guidelines issued by the American Academy of Pediatrics [2] (AAP). New evidence has however been collected in recent years concerning new therapeutic options [3, 12]. Nonetheless, the small number of treatments effective for bronchiolitis emphasizes the importance of prevention in decreasing the impact of this disease.

## Diagnosis of bronchiolitis

Bronchiolitis is a viral disease the diagnostic workup of which is divided into two main phases: clinical diagnosis of bronchiolitis and etiological diagnosis.

Clinical diagnosis of disease and its severity is rooted in the clinician's interpretation of the constellation of characteristic findings, and is independent from any specific clinical features and diagnostic tests. Children with acute bronchiolitis in fact may present with a wide range of clinical symptoms, from mild respiratory distress to incipient respiratory failure [13]. Therefore, a careful physical examination (rhinorrhea, cough, crackles, wheezing, dyspnea, polypnea, feeding difficulties, apnea, lethargy) combined with the collection of clinical history (to assess the course of the disease and to capture any high risk conditions) are warranted as the cornerstone of the diagnosis of bronchiolitis, which in this document makes reference to children aged up to 12 months. Diagnostic criteria for the disease include, but are not limited to, the following [13, 14]:

-  Onset with rhinorrhea and/or upper respiratory tract infections
-  First episode of respiratory distress associated with: crackles and/or wheezing, use of accessory muscles or lower chest wall retractions, low O_2_ saturation levels, high respiratory rate relative to age, skin color changes, nasal flaring, fever
-  Exposure to persons presenting with upper respiratory tract viral infections
-  Presentation during epidemic season

Elements such as gestational age or birth age and progression of clinical signs must be carefully assessed.

Despite not being recommended in clinical practice [2, 3], an etiological diagnosis may be useful especially in the hospital setting to avoid use of antibiotics (once viral origin has been demonstrated) and to decrease risk of hospital infections by means of “cohorting” (practice by means of which all children suffering from viral bronchiolitis are grouped together in order to avoid contact with susceptible patients).

Tests to identify presence of the virus may moreover have an epidemiological significance. The virus may be isolated by the following methods, which aim at identifying either the virus (through genome detection) or its antigens:

1. Antigen detection (immunofluorescence, enzyme immunoassay). These are the so-called “Rapid antigen-detection tests” with an 80–90% sensitivity, which might yield false negatives in infants younger than 3 months of age. As the quantity of antigen required by these tests is rather significant, their sensitivity is in direct proportion to the quality of the sample collected. The sample may be collected by nasal lavage or swab (using flocked swabs). Results are ready after a short interval (30–60 minutes).
2. Genome detection (*In situ* hybridization, traditional or real-time polymerase chain reaction (PCR)). PCR is the gold standard diagnostic test in consideration of its 93-100% sensitivity and its 64-100% specificity.

Although both techniques are extremely valid, antigen assays are usually used in routine practice since PCR assays are more expensive and not always available.

Collecting samples: the sample to be analyzed must be collected within 6–7 days after the onset of the infection; if this is not the case, positivity will decrease together with the viral load [15].

Nasal swabs (using flocked swabs) and nasal lavage (using at least 3 mL of saline solution) followed by nasopharyngeal aspirate provide the best specimens in terms of most effective detection of etiological agents [16, 17].

Neither laboratory tests or radiological exams are usually indicated for the routine workup of infants with bronchiolitis [2, 3].

## Pediatric primary health care assistance

It is important to point out that the milder forms of bronchiolitis may be adequately managed in the outpatient setting by primary care pediatricians, thus limiting hospital admissions. In the outpatient setting the child’s general clinical conditions must be assessed, together with his/her ability to feed, heart rate, respiratory rate, oxygen saturation (measured by pulse oximetry with specific sensors for infants), the presence of any risk factors and family compliance. If hospitalization is not indicated, the parents must be instructed when to ask for primary care pediatricians or the Emergency Room reassess the child; moreover, parents’ compliance with indications, as well as their evaluation ability and the absence of any difficulties that might hinder their return for a possible reassessment must be ascertained. Support and pharmacological therapies for outpatients management are illustrated in the chapter “Treatment of Bronchiolitis”.

## Indications to hospitalization

The admission status must be assessed on a case-by-case basis, as there have been no findings from physical examination consistently associated with outcomes of bronchiolitis [13].

Hospitalization is warranted based on the following conditions:

-  O_2_ saturation persistently lower than 90-92%, entity of respiratory distress, presence of apnea. In patients with congenital heart disease or BPD the need for oxygen therapy must be determined relative to habitual transcutaneous saturation measured at the child’s baseline status of well-being and not relative to the levels in healthy children (e.g. O_2_Sat might be 88% in a Fallot patient).
-  Dehydration
-  Moderate–severe bronchiolitis (Table 1 and Table 2) [18]

**Table 1:** **Normal respiratory rate and heart rate values**

Respiratory rate	Heart rate
**(normal values)**	**(normal values)**
< 2 months: <60/min	0-12 months: < 160/min
2-12 months: <50/min	1-2 years: < 120/min
>1-5 years: < 40/min	2-8 years: < 110/min
6-9 years: < 30/min	
10-14 years: < 20/min

**Table 2:** **Assessment of bronchiolitis severity** [18]

	***Mild***	***Moderate***	***Severe***
**Respiratory rate**	Normal to slightly increased	Increased	Markedly increased compared to normal values (refer to Table 1)
**Respiratory effort**	Mild chest wall retraction	Tracheal tug	Marked chest wall retraction
		Nasal Flare	Nasal flare
		Moderate chest wall retraction	Grunting
**Oxygen saturations**	No supplemental oxygen requirement, O_2_ saturations > 95%	Saturations 90-95%	Saturations < 90%, may not be corrected by O_2_
**Feeding**	Normal to slightly decreased	50-75% of normal feeds	< 50% of feeds, unable to feed
**Apnoea**	Nil	May have brief episodes	May have increasing episodes

Other important factors to take into consideration are:

-  Prematurity, gestational age < 37 weeks or birth age < 6–12 weeks [2]
-  Responsivity and alertness
-  Decreased hydration and feeding (<50% of usual fluid intake in preceding 24 h [19])
-  Social factors: distance from the hospital, access to means of transportation or communication, parents’ collaboration and reliability in terms of managing the child at home or of understanding the clinical signs associated with the worsening of the disease
-  Environmental factors: exposure to cigarette smoke, humid or cold dwelling, crowded dwelling
-  Presence of pre-existing risk factors, which include: BPD, cyanogenic congenital heart disease and/or congenital heart disease associated with pulmonary hypertension, prematurity [2]; other diseases which might complicate the presentation of acute respiratory infections include immunodeficiency, airway malformation, severe neurological deficit [2, 7], cystic fibrosis. In presence of any of these factors is necessary to consider hospitalizing the patient and/or possible arranging for the patient to be closer to a hospital with a pediatric intensive care unit.

After having be assessed in the Emergency Room, if hospitalization is not indicated, parents must be instructed to have the child reassessed by the primary care pediatrician or by pediatricians of the local Emergency Room. Compliance of parents must be ascertained as well as the absence of any difficulty which might prevent them from returning to the hospital.

Whenever present, admission to the Short-Stay Observation Ward (*Osservazione Breve Intensiva -OBI*) is warranted in order to follow clinical progression for patients for whom hospitalization is not immediately indicated.

## Indication to transfer to intensive care unit

Children with severe bronchiolitis must be transferred to the Pediatric Intensive Care Unit, based on the following conditions:

-  Respiratory failure which requires mechanical ventilation support (Continuous positive airway pressure (CPAP) support should be managed as sub intensive assistance).
-  Apnea with desaturation
-  Severe impairment of general conditions

## Treatment of bronchiolitis (outpatients setting/emergency room/hospital)

The basic management of bronchiolitis is based on treatments that assure the patient is clinically stable, well oxygenated and well hydrated [13], with repeated clinical assessment. Lacking a specific etiological treatment, therapy for bronchiolitis includes supportive and pharmacological therapies to control respiratory and systemic symptoms [2, 11, 19] (Table 3).

### Supportive therapy

#### Oxygen therapy

In accordance with the AAP guidelines [2], supplemental oxygen should be administered if O_2_ saturation levels are persistently below 90% at ambient air (SIGN [19] guidelines instead suggest starting O_2_ therapy in presence of saturation levels lower than 92%). It is important that O_2_ saturation be measured correctly by pulse oximetry (using pediatric probes, with nasal aspiration prior to measurement, avoiding measurement when child is moving limbs; reading is to be performed when plethysmograph wave intensity is broad and stable, while measurement is not reliable in case of peripheral vasoconstriction or of hypothermia of the limbs). Humidified O_2_ may be administered by means of nasal prongs or mask. O_2_ saturation must be monitored throughout the entire duration of O_2_ supplementation and discontinued when O_2_ saturation is about 93%, in presence of a stable improvement of symptomatology, and if the child has resumed intake of fluids and feeds. In subjects with risk factors for severe respiratory failure (congenital heart diseases, bronchopulmonary dysplasia, prematurity) monitoring should continue even after weaning off oxygen therapy until the patient is completely stabilized, in order to capture onset of further episodes of hypoxia which might occur in these children [2].

High flow oxygen therapy with humidified and heated oxygen (High Flow Nasal Cannula, HFNC) is a new noninvasive ventilation support modality that generates positive pressure in the pharynx and decreases workload of respiratory muscles [20, 21]. Preliminary studies suggest that the use of high flow heated and humidified oxygen up to 2 L/kg/min (maximum of 10 L/min), may rapidly improve oxygen saturation in infants suffering from bronchiolitis [22]. An advantage of this respiratory support technique moreover lies in the fact that it may be used also in pediatrics wards, and not only in Pediatric Intensive Care Units [23]. Studies are currently ongoing to assess efficacy of HFNC ventilation support relative to major outcomes, such as length of hospital stay and need for transfer to Intensive Care Unit.

Oxygen therapy may be discontinued and discharge may be taken into consideration for children presenting with O_2_ saturation levels ≥ 93-94% [19], who have resumed feeding and who experience minimal respiratory distress [2].

#### Nebulized hypertonic saline solution

The use of nebulized hypertonic 3% saline solution during bronchiolitis has been well documented in recent years [24–27], and shown to be effective in improving clinical scores and length of hospital stay. However further large-scale studies are needed before conclusions can be made.

Nebulized hypertonic saline has been used both in the mild and in the moderate-severe forms of the disease in the outpatient settings and in hospital [24]. The mechanism of action of nebulized hypertonic saline solution seems to be linked with a decrease of airway edema, improved ciliary clearance of mucus and decreased respiratory secretion viscosity [28]. Aerosol administration of hypertonic 3% saline solution is effective and well tolerated (uncomplicated by onset of bronchospasm) even when not administered in association with bronchodilators [29]. According to available studies, examined in a recent Cochrane [24], hypertonic saline has been administrated every 2 hours in hospitalized patients during the initial treatment phase (the first 6–8 hours), and every 4–6 hours thereafter. At the Emergency Room, instead, 2–3 consecutive doses have been administrated even less than every 2 hours (30 minutes). The most widely used saline solution volume in these studies is 4 mL. No significant adverse events related to hypertonic saline inhalation, such as tachycardia, hypertension, pallor, tremor, nausea, vomiting and diarrhea were observed in these trials. The inhalation therapy was administered via pneumatic nebuliser in most of the studies, but further studies are required to compare different nebulisers [24].

#### Nasal aspiration

International guidelines currently in use [2, 19, 30] recommend superficial aspiration of the upper airways especially in younger children in order to improve airway patency and to ease feeding difficulties. Deep airway aspiration in presence of bronchiolitis is on the other hand not recommended, as one study demonstrated that this procedure associates with an increase of the length of hospital stay [31].

#### Feeding and hydration

In presence of bronchiolitis, maintaining an adequate hydration may be difficult because of the concomitance of fever, tachypnea, and difficulties in taking feeds and liquids [2, 19]. Breastfeeding or feeding with baby bottle should be encouraged for all breastfed infants who are able to feed, in order to replace fluid losses [14]. Feeding 2–3 hourly with decreased volumes may be helpful to prevent the risk of inhalation [18]. In case of moderate or severe bronchiolitis associated with difficulties in oral intake of liquids, rehydration may be administered intravenously or by nasogastric tube [32]. According to a recent study [33] there are no significant differences between the two modalities of liquid administration in terms of length of hospital stay, transfer to intensive care and need for mechanical ventilation, although the placement of the nasogastric tube has been shown to be the easier maneuver.

Fluid and electrolyte balance must be accurately monitored, especially when administering fluids intravenously to avoid onset of hyponatremia and/or inappropriate secretion of ADH, complications which may occur during bronchiolitis [2] and worsen prognosis of this disease [34].

#### Chest physiotherapy and ambient humidification

While evidence supporting usefulness of ambient humidification is insufficient [35], it has been demonstrated that chest physiotherapy (vibration and percussion techniques) is ineffective during the acute phase of bronchiolitis [36].

### Pharmacological therapy

#### Beta2-agonists

As per international guidelines [2, 19], two recent systematic reviews [37, 38] have confirmed that inhaled beta agonists are not effective for bronchiolitis, in that they do not improve oxygen saturation, do not decrease need for and length of hospital stay nor reduce overall duration of symptoms. However, as a favourable response may be observed in a number of cases, a single therapeutic trial with salbutamol by aerosol may be considered, in particular in children with a family history of allergy, asthma and/or atopy. This therapy should not be continued lacking a documented clinical improvement (decrease of respiratory rate and/or respiratory effort) 15–30 minutes after the trial inhalation treatment [14].

#### Nebulized adrenaline

A recent meta-analysis reviewed available studies on the use of nebulized adrenaline for bronchiolitis and concluded that it might be effective in decreasing need for hospitalization in children presenting to the emergency room, whilst it was not noted to be effective in decreasing length of hospital stay in hospitalized children [38]. A recent Norwegian multicenter study confirmed that administering nebulized adrenaline to hospitalized children is not more effective than nebulized 0.9% saline solution in terms of length of hospital stay, clinical score and need for supportive therapy (O_2_, ventilation support) [39]. Because of lack of studies, short duration of action, and potential adverse effects, home use of nebulized adrenaline is not recommended by international guidelines [2, 19], which suggest that a bronchodilator (salbutamol) trial would be more appropriate in the office/clinic setting. Further studies are necessary to evaluate the early use of nebulized adrenaline.

#### Systemic and nebulized steroids

Large reviews performed over the years concerning the use of both systemic and inhalation steroids for bronchiolitis [38, 40–42], have shown that these drugs are not effective in decreasing incidence and duration of hospitalization or in improving short– and long-term prognosis. This is probably due to the fact that bronchiolitis is characterized by a profound neutrophilic airway inflammation. There is no consistent clinically relevant association of therapy with systemic or inhaled glucocorticoids and improved outcomes in either outpatient or inpatient settings [41].

#### Association of adrenaline and steroids

A multicenter randomized study involving children aged between 6 weeks and 1 year [43] demonstrated that treatment with adrenaline via aerosol and high-dose dexamethasone per os administered at the Emergency Room reduces risks of hospitalization during the first 7 days of the disease, suggesting a synergism of the two medicinal products. However, the study had not been designed to assess the effect of the combination therapy and was the subject of controversy because of the high steroid dose used.

#### Antibiotics

The AAP guidelines [2] suggest that antibiotics be used only in cases of bronchiolitis with bacterial co-infection, as documented by culture or molecular tests, and in children with severe bronchiolitis admitted to the Intensive Care Unit [44]. The habitual use of antibiotics in case of bronchiolitis must be avoided because of the risk of side effects, significant costs and possible development of antibiotic resistance.

It has been suggested but not been confirmed that macrolides may have an anti-inflammatory and immunomodulator effect in presence of bronchiolitis [45] and therefore they are not to be used for this purpose.

#### Antivirals

Current guidelines do not recommend use of ribavirin as therapy for bronchiolitis [2, 19, 30]. A recent review [46] confirmed that currently available data are insufficient to recommend the use of ribavirin in the treatment of bronchiolitis.

#### Other therapies

Other pharmacological and supportive treatment such as montelukast, DNase, inhaled furosemide, methylxanthine, have been assessed in the treatment of bronchiolitis in pediatric subjects.

Currently available studies do not show results concerning efficacy of montelukast in the treatment of bronchiolitis and prevention of recurrent post-bronchiolitis wheezing [47–52].

There is no scientific evidence supporting efficacy of inhaled DNAse in improving clearance of respiratory secretions [53]. The use of methylxanthine (caffeine and theophylline) has also been proposed to reduce apnea in presence of bronchiolitis [54, 55], however the lack of precise epidemiological data concerning the incidence of central sleep apnea does not allow to confirm its usefulness [56].

Table 3 summarizes the evidence relating to therapy for bronchiolitis.

**Table 3:** **Treatment of bronchiolitis**

Supportive therapy		
**Oxygen therapy**	If O_2_Sat < 90-92%	AAP, 2006 [2] SIGN 2006 [19]
**Nebulized 3% hypertonic saline**	Safe and effective	Cochrane (Zhang) 2013 [24]
	Recommended	Morawetz, 2011 [25]
		Ralston, 2011 [26]
		Kuzik, 2007 [27]
		Mandelberg 2010 [28]
**Respiratory physical therapy during acute phase of disease**	Not effective	Cochrane (Roquè), 2012 [36]
**Environment humidification**	Insufficient evidence	Cochrane (Umoren), 2011 [35]
**Pharmacological therapy**		
**Nebulized Beta 2-agonists**	Not effective (the possibility of a therapeutic trial of salbutamol is contemplated)	Cochrane (Gadomski), 2010 [37]
		Hartling, 2011 [38]
**Nebulized adrenaline**	Decreases hospitalizations in patients presenting to ER	Hartling 2011 [38]
		Skjerven 2013 [39]
**Nebulized and systemic steroids**	Not effective	Cochrane (Fernandes) 2013 [41]
		Hartling, 2011 [38]
		Blom, 2010 [40]
		Corneli, 2007 [42]
**Nebulized adrenaline + systemic steroids**	Further studies are necessary	Plint, 2009 [43]
**Antibiotics**	Only in selected cases (bacterial co-infection/pre-existing diseases)	Cochrane (Spurling) 2011 [44]
		Pinto, 2012 [45]
**Ribavirin**	Only in selected cases (severe forms/pre-existing diseases)	Cochrane (Ventre) 2010 [46]
**Nebulized DNase**	Not effective	Cochrane (Enriquez) 2012 [53]
**Montelukast**	Apparently not effective	Zedan, 2010 [47]
		Amirav 2008 [48]
		Bisgaard 2003 [49], 2008 [50]
		Proesmans, 2009 [51]
		Kim, 2010 [52]

## Criterias for discharge

-  Sustained autonomy from any kind of respiratory support and O_2_ saturation levels > 92-94% at ambient air
-  Stabilization of clinical presentation
-  Adequate oral intake of fluids and feeds (>75%) [19]
-  Adequacy of the family unit in terms of providing monitoring and possible continuation of therapy at home
-  Possibility, if necessary, of obtaining pediatric health care assistance locally.

## Prevention

Prevention of bronchiolitis in pediatric subjects basically includes environmental prophylaxis to decrease transmission of respiratory infections and, specifically for RSV bronchiolitis, pharmacological prophylaxis with administration of humanized monoclonal antibodies (palivizumab) during the epidemic season in particular categories of children at risk. There is no vaccine against RSV available.

### Environmental prophylaxis

Environmental prophylaxis is indispensable to decrease diffusion of the virus in the hospital setting, at outpatient clinics, and at home, since the virus is easily spread by airborne transmission, via saliva droplets, and through contact with contaminated objects and surfaces (hands, garments, toys, medical instruments, kitchen utensils etc.) on which the virus may deposit and remain active for several hours [2, 57].

Compliance with hygiene measures for hands and contaminated surfaces has been well established to be an easy, non-costly and very effective technique in reducing epidemic diffusion of RSV and other respiratory viruses [58], with RSV hospital infection rates dropping from 4.2 to 0.6-1.1% in children aged < 2 years and from 34.8% to 2.1-3.3% in children presenting with congenital heart diseases [59].

Environmental prophylaxis is to be carried out complying with the indications summarized below [2, 19, 60]:

-  frequent handwashing and decontamination of hands using alcohol solutions, cleaning of solid surfaces using water and disinfectants (alcohol based or antibacterial detergents are to be privileged in case of contamination with abundant organic material) or sodium hypochlorite. In the hospital or outpatient clinic setting, multiple-use medical equipment (e.g. stethoscopes) must also be decontaminated.
-  sharing kitchen utensils and personal effects must be avoided for subjects with known infection.
-  subjects presenting with respiratory infection symptoms are recommended to cover nose and mouth with masks and to wash hands prior to touching anything.
-  attendance at community settings or environments where risks of contact with infected persons are high, is to be reduced to a minimum as far as possible for young children and in particular those belonging to the aforesaid risk categories.
-  avoid exposure to secondhand cigarette smoke since this increases risks of developing respiratory infections [2].

In addition to those described above, several indications have been proposed with a view to limiting hospital RSV infections in the wards where high risk subjects are hospitalized [2, 19, 61, 62]:

-  use of rapid tests to identify RSV-positive patients and to cohort them apart from RSV negative patients
-  use of disposable gloves and white coats for healthcare providers who are in contact with the patient
-  use of barrier devices in case of maneuvers which put into contact with respiratory secretions (feeding, airway aspiration, aerosol therapy)
-  decrease of the number of persons entering in contact with high risk patients during the epidemic period.
-  Compliance with these recommendations has led to a 39–50% decrease of RSV hospital infections [63, 64].

### Pharmacological prophylaxis

Palivizumab is a humanized monoclonal antibody that binds the F protein on the surface of RSV thus blocking the fusion of the virus membrane with the target cell membrane. The pharmacokinetic properties of palivizumab have been analyzed in preterm infants with a gestational age of ≤35 weeks (wGA) and birth age ≤6 months [65], and in children presenting with BPD aged ≤2 years [66]. Results have shown that palivizumab is tolerated well and that IM administration of a 15 mg/kg dose once a month helps maintain average antibody serum concentrations sufficient to prevent RSV infection. In consideration of its half-life of approximately 20–30 days, clinical studies findings and guidelines issued by numerous pediatric scientific societies suggest that monthly administration of palivizumab is indicated from the start of the epidemic period for up to 5 doses [67]. Efficacy and safety of palivizumab have been documented also by 2 multicenter randomized studies conducted on preterm infants younger than 6 months and preterm infants younger than 2 years and presenting with BPD respectively [68], as well as on children with hemodynamically significant congenital heart diseases [69]. These studies have demonstrated efficacy of prophylaxis in decreasing the number of hospitalizations and reducing length of hospital stay without any findings of serious adverse events. Pursuant to these studies, the FDA has granted authorization to marketing of the drug for the aforesaid risk categories.

Numerous scientific societies have issued indications concerning categories of subjects who should receive treatment, during the epidemic period, relative to gestational age and birth age [67, 70–72]. There is substantial consensus concerning treatment of subjects presenting with specific congenital heart diseases (please refer to the chapter “Children with heart disease”), subjects with BPD that required therapy during the six months preceding the epidemic season (through the second birthday) and of preterm children with ≤ 32 wGA (until the first birthday). As regards the category of children with 33 to 35 wGA, indications arising from epidemiological studies conducted in various countries differ in part regarding duration of prophylaxis and presence of identified risk factors. For subjects of 33 to 35 wGA + 6 days, prophylaxis is recommended in the epidemic period during the first 90 days of life (therefore up to 3 doses) in presence of at least 1 of the 2 risk factors indicated: attendance of the child in a community setting and/or presence of one or more cohabitees younger than 5 years. In Italy, indications issued by the Italian Society of Neonatology (SIN) concerning prophylaxis date back to 2004 [72], however there is no unanimous consensus concerning application of said indications in subjects with 33–35 wGA. The SIN Board has recently appointed a multidisciplinary task force to update current recommendations based on the new international and national scientific evidence; to this end, the currently ongoing analysis of the data generated by the Italian multicenter study sponsored by SIN in collaboration with the Italian National Research Councils (CNR) will in all likelihood yield useful elements [73], thus allowing to identify risk factors for RSV bronchiolitis hospitalization in infants with ≥ 33 wGA and to define the birth age cut-off prior to which prophylaxis is the most useful. The intersociety document will be updated based on indications generated by the study.

### Vitamin D

Recent studies have demonstrated that infants with vitamin D deficiency at birth face a higher risk of developing RSV infection during the first year of life [74] and that vitamin D supplementation allows to decrease risks of viral respiratory tract infections [75]. These data suggest that vitamin D supplementation for pregnant women and for infants might be a useful strategy in preventing viral respiratory infections causing bronchiolitis [74, 75]. Currently recommended doses are 400 IU/day for children younger than one year and 600 IU/day for children older than one year. Further interventional studies are necessary to assess the true role played by vitamin D in preventing the disease.

## Later respiratory outcomes of bronchiolitis

RSV infections in infants may interfere with normal immunological and pulmonary development and might correlate with an increased incidence of recurrent bronchospasm in preschool-aged infants, and with asthma and decreased respiratory function in school-aged children [9, 10]. Recent studies have demonstrated that also when caused by other respiratory viruses, such as Rhinovirus, bronchiolitis associates with a greater risk of recurrent wheezing [76]. Post-bronchiolitis syndrome is more frequent in severe cases of RSV bronchiolitis which required hospitalization. Recent Northern European follow-up studies conducted throughout adult age (18 and 30 years) have demonstrated that up to 30 – 40% of subjects who had been previously hospitalized because of bronchiolitis presents with asthma and uses antiasthmatic drugs [77].

However, it is as yet unclear whether the viral infection is the direct cause of the subsequent development of asthma (by damaging the airways) or if it instead acts by “unmasking” asthma (or bronchial hyperactivity) in genetically or structurally predisposed children. A recent randomized, placebo-controlled, double-blind study [78] has demonstrated that prophylaxis with Palivizumab in healthy premature children (33–36 wGA) is able to decrease by 61% the number of days with bronchospasm during the first year of life, thereby supporting the hypothesis of a direct damage caused by the virus. No similar studies are available in children born at full term.

## Special populations: a dilemma

It is well known that RSV infections may be severe and even potentially fatal in specific populations of high risk newborns and infants.

These groups of patients were traditionally identified mainly based on the “prematurity” factor, building on the axiom that “the greater the prematurity, the greater the risk of developing severe RSV disease”. In actuality, in recent years awareness has been reached to the effect that significant risks of severe RSV infections may be detected in infants and children suffering from a number of rare medical conditions for which prematurity might be only a possible risk cofactor [7]. In such children, risk is not associated with immaturity of the respiratory tract but rather with the presence of specific anatomical, functional, immune and pathophysiological conditions such as to generate major risks of severe disease in presence of bronchiolitis [7, 79–81].

Children with congenital neuromuscular diseases might have poor residual functional capacity, ineffective coughing because of respiratory muscle weakness, decreased glottal closing ability, decrease of spontaneous physiological movements with reduction of normal ventilation redistribution, and high prevalence of gastroesophageal reflux with consequent repeated inhalation and aspiration syndromes [82].

The latter issue is of course present also in patients presenting with any of a number of malformation syndromes or sequences (e.g. Pierre-Robin, CHARGE, Jeune syndrome etc.) in which normal interactions between swallowing, respiration and food bolus transit are affected [7, 79–82].

Another category of high risk children is represented by patients with cystic fibrosis. The RSV infection in these children has been documented to accelerate the respiratory function decline which they will be facing over the years, causing a more rapid onset of wheezing and bacterial superinfections. The known close correlation between RSV and *Pseudomonas aeruginosa* is crucial in this process: indeed, proliferation and epithelial adhesion of the latter pathogen is facilitated by the concomitant presence of RSV [7, 83]. The preliminary results of a recent study suggest a trend towards less RSV-related hospitalizations in children with cystic fibrosis treated with palivizumab, however the study involved few patients and results are to be considered as exploratory [84].

Patients with Down syndrome have long been considered to be at high risk for RSV exclusively because underlying severe heart disease is often present. In actuality, according to cohort studies conducted in the Netherlands and in Israel collecting data starting at birth have demonstrated that the incidence of RSV-related hospitalizations is of approximately 10% (about 3–4 times higher than in the normal population) in all Down subjects, also in children without congenital heart disease, as associated with their documented state of relative T lymphocyte immunodeficiency [85]. This finding suggests that specific prophylactic measures are warranted in these children during their first two years of life [85–87].

Finally, a mention must be made of the composite group of newborns and infants who might develop a severe RSV disease because of their reduced immunological competence [88–90]. Response of the immune system against RSV, as is known, involves the innate epithelial response (at the level of nasal mucosa), secretory antibody response (specific IgG, at the level of the upper airways limiting passage of the virus to the lower airways), and, finally, the T lymphocyte response, which is responsible for viral clearance and resolution of the RSV infection.

Patients with congenital immunological syndromes, primary or secondary immunodeficiency (such as bone marrow or solid organ transplant recipients, patients with SCID, Di George or Wiskott-Aldrich syndromes, patients with neonatal HIV, etc.) demonstrate a marked inability to clear the virus, to prevent its replication or to limit its potential to determine pulmonary damage [7]. Thus, these patients are destined to be RSV carriers for a longer period of time, with higher viral loads and with greater virulence. In this respect, patients at greater risk are precisely those with immunodeficiency affecting the T lymphocyte response, while a decrease in effectiveness of the B-mediated antibody immune response is not as severe [89, 90].

Unfortunately, methodologically valid scientific documentation supporting safety and efficacy of anti-RSV prophylaxis with palivizumab in these populations is still lacking. In consideration of the low incidence of these conditions, randomized studies are not feasible or are difficult to propose, and this is a limitation which is not expected to be overcome in the foreseeable future.

However, experts do recommend that prophylaxis with palivizumab be considered in children presenting with neurological or neuromuscular diseases, genetic syndromes receiving oxygen therapy or ventilation at home during the first and second year of life and during epidemic periods.

## Children with heart diseases

Approximately 8 children out of 1000 live births suffer from congenital heart disease (CHD) at birth, and approximately 50% of these children present also with hemodynamically significant impairment requiring medical and/or surgical and/or invasive cardiological therapeutic measures, often during the very first years of life. Children with hemodynamically significant congenital heart diseases are at higher risk of possibly severe and potentially fatal complications arising from lower respiratory tract RSV infections, as well as of hospital-acquired RSV infection [91–94].

In 2004, Cabalka [95] pointed out that young children suffering from hemodynamically significant CHD associated with pulmonary hyperflow, pulmonary congestion and left/right shunt, or with cyanosis, present with multiple and complex cardiocirculatory and respiratory pathophysiological risk factors that impede an adequate compensatory response to any intercurrent disease. These infections may complicate management of heart failure or of cyanogenic heart diseases and/or induce or worsen pulmonary hypertension, ultimately making it necessary to postpone an already scheduled surgical procedure or jeopardizing the outcome of heart surgery in the post-surgery phase. The RSV hospital acquired infection is an unfavourable perioperative prognostic factor in children presenting with CHD requiring surgery [96]. Moreover, the virus may cause cardiac complications in hospitalized children, such as sinoatrial block, tachyarrhythmias, atrioventricular block of varying entity, pericarditis and myocarditis [97]. The efficacy and safety of palivizumab in children presenting with hemodynamically significant congenital heart diseases has been documented by a large multicenter, randomized, double-blind prospective study comparing palivizumab and placebo [69]. This study has confirmed the efficacy of prophylaxis in decreasing the number of RSV-related hospital admissions (-45% globally; -58% of children with non-cyanogenic heart disease; -29% of children with cyanogenic heart disease), as well as length of these hospital stays (-56%) without any unfavourable effect on surgical outcomes. The Italian Society of Pediatric Cardiology (SICP) [98] also drafted in 2006 indications concerning prophylaxis with palivizumab in patients suffering from childhood congenital or acquired “severe” heart diseases, in order to better define the concept of “hemodynamically significant” heart disease and therefore render the cost–effectiveness ratio of prophylaxis more acceptable.

The following categories of children are to be considered as suffering from “hemodynamically significant” congenital heart disease and as eligible for prophylaxis with Palivizumab:

-  children aged ≤ 2 years at the beginning of the RSV epidemic season presenting with either congenital or acquired hemodynamically and clinically relevant heart diseases and cardiomyopathy such as:
  - Heart diseases with significant pulmonary hyperflow (cardiomegaly and/or polypnea and/or radiographic pulmonary congestion and/or failure to thrive and/or medical therapy and/or recurrent lower respiratory tract infections)
  - Cyanogenic heart disease prior to surgical procedure or after a palliative procedure
  - Primary or secondary pulmonary hypertension (equal to or greater than 50% of systemic pressure)
  - Heart diseases associated with pulmonary venous congestion
  - Dilated cardiomyopathy on medical therapy to treat heart failure
  - Heart diseases after surgical or other procedures until medical therapy for heart failure is discontinued
  - Recipients of heart transplant

Administration modalities and doses are comparable to those for other categories at risk.

In children undergoing surgery with extracorporeal circulation and who continue to be at risk for RSV infection, a post-surgery dose of palivizumab may be administered as soon as the child is considered to be clinically stable (because of a 58% decrease in serum levels of palivizumab that was observed after cardiopulmonary bypass surgery).

Subjects presenting with the following conditions are not eligible for prophylaxis with Palivizumab:

-  mild or moderate heart diseases which do not require medical treatment, surgery or other kind of intervention during the first 24 months of life (for example small uncomplicated inter-atrial or intraventricular defects, small patent ductus arteriosus, mild aortic coarctation, mild–moderate pulmonary or aortic stenosis, partial atrioventricular canal defect)
-  children presenting with mild cardiomyopathy which does not require therapy
-  children with CHD which was adequately corrected by surgery, without any hemodynamically significant sequelae (please refer to eligibility criteria).

## Conclusion

Bronchiolitis is the leading cause of hospitalization in children younger than one year, and at times requires admission to the intensive care unit. Children younger than 3 months or children presenting with risk factors such as prematurity, bronchopulmonary dysplasia, congenital heart diseases and immunodeficiency and other malformation syndromes or diseases are at particular risk of severe and potentially fatal bronchiolitis. Primary prevention is feasible by carefully implementing environmental prophylaxis. In subjects at greater risk, prophylaxis with Palivizumab against the most common responsible infecting agent RSV, is indicated during the epidemic period, as recommended by scientific societies. Assistance provided to children with bronchiolitis is optimized by implementing shared strategies issued based on the most recent and robust scientific evidence concerning hospitalization criteria, diagnosis, monitoring, treatment. This will have a major favorable effect on these children, their families and on direct and indirect costs generated by the disease and by possible later outcomes. This Document will be accordingly and consistently updated as new evidence arises in this field.
